# Physical Education Pedagogies Built upon Theories of Movement Learning: How Can Environmental Constraints Be Manipulated to Improve Children’s Executive Function and Self-Regulation Skills?

**DOI:** 10.3390/ijerph16091630

**Published:** 2019-05-10

**Authors:** James R. Rudd, Laura O’Callaghan, Jacqueline Williams

**Affiliations:** 1 School of Sport Studies, Leisure and Nutrition, Liverpool John Moores University, Liverpool L176BD, UK; L.A.OCallaghan@2017.ljmu.ac.uk; 2 Institute for Sport and Health, College of Sport and Exercise Science, Victoria University, 8001 Melbourne, Australia; Jacqueline.Williams@vu.edu.au

**Keywords:** cognition, physical activity, movement competence, skill acquisition, motor development, primary education

## Abstract

Physical education in schools has been marginalised across the globe, and as a result, children are missing out on opportunities to develop and acquire the foundation skills needed to lead a physically active life. The squeeze on physical education in schools, particularly in some western countries (United Kingdom, Australia and America), has been justified on the grounds that core subjects such as English and mathematics need more curriculum time, as this will lead to higher cognitive and academic performance. The aim of this paper is to highlight how physical education lessons in early childhood, underpinned by either of two major theories of motor learning, can support teachers in the creation of learning environments, as well as guide their pedagogical practice to facilitate children’s development of key cognitive skills, in particular executive function and self-regulation skills. These skills are crucial for learning and development and have been found to be a higher predictor of academic achievement than IQ. They also enable positive behaviour and allow us to make healthy choices for ourselves and others, therefore providing further evidence that the development of movement skills has the potential to secure positive attitudes and outcomes towards physical activity across the lifespan.

## 1. Introduction

In spite of international [1] and national [2] policy-led campaigns, physical inactivity continues to cost the United Kingdom (UK) economy over £7 B each year [3] and, internationally, an estimate in excess of £51.5 B a year [4]. The UK government and World Health Organisation recommends that children and young people should take part in moderate to vigorous physical activity (PA) for a total of 60 min per day in order to achieve the associated health benefits [5,6], and yet, in 2012, as few as 20% of young people were meeting the recommended guidelines [7].

Physical education (PE) is deemed to be the subject on the school curriculum that enables children to develop foundational movement skills and builds the knowledge and understanding needed to lead physically active lifestyles [8]. However, it has progressively become marginalised within the primary school timetable and is commonly viewed as a lower priority than core subjects, such as mathematics [9,10], which, typically, are prioritised by schools, because government policy has introduced national standardised tests in these subjects [11,12,13]. Results from these tests are used as a means of performance management for schools [14], the justification being that these core subjects provide a curriculum that imbues cognitive knowledge and enables competition with local, national and international competitors [15].

This position may, however, be short-sighted, as there is a growing body of evidence that demonstrates a link between PA and cognition, as regular PA leads to better circulation and an improved oxygen supply to the brain [16]. Further biological and psychosocial hypotheses have sought to explore the characteristics of PA, which influence the experience-dependent plasticity of the brain regions responsible for children’s cognitive skills [17]. A majority of the research to date has focussed on the positive influence of PA dose and fitness level on cognition [18]. Structured PA focussing on movement skill development and a high level of challenge has been found to have a stronger impact on cognitive function than computerised “brain training” programs. For instance, Moraeu et al. [19] found that learning complex movements facilitates greater improvements to cognitive abilities compared with computer-based cognitive training intervention. Longitudinal evidence highlights that possessing a high level of movement skills during early childhood predicts higher cognitive development and academic achievement [20,21,22,23]. Despite the aforementioned benefits of high movement competence, there is evidence of a secular decline in some movement skills and abilities [24]. In particular, children from areas of high deprivation have less developed movement skills than their peers from more affluent areas [25,26]. Children living in deprived areas are also more likely to be disadvantaged in terms of their long-term health and their social and economic wellbeing [27]. Consequently, these children are more at risk of accumulating early negative experiences or being exposed to environmental deprivation that exerts harmful effects on the developing brain, with adverse implications for movement skill and wider development [28,29]. The low prevalence of movement competence amongst children from areas of high deprivation indicates that these children should be prioritised for opportunities to engage in meaningful experiences in PE that foster movement competence.

Arguably, the most important cognitive skills for academic success are a family of higher order skills termed executive function (EF). The three core skills within this family are inhibitory control, working memory and cognitive flexibility [30]. EF is considered to be more predictive of academic achievement than IQ and affects a child’s level of engagement within their environment [30,31]. Similar to movement competence, the development of EF is protracted throughout childhood and into adolescence [31]. This reflects the extended period of development of brain regions associated with EF, such as the dorsolateral prefrontal cortex and cerebellum (both also linked to movement skill learning and performance) [32], and provides the opportunity for environmental enrichment (resulting in experience-dependent plasticity) throughout childhood [17]. EF is intertwined with the development of the self-regulation required for a child to be able to coordinate goal-direction action. Finding alternative actions will require self-regulation to maintain optimal arousal (physical, emotional and cognitive), and inhibitory control will overcome pre-potent responses, such as resisting the temptation to kick a ball when the teacher is giving an instruction [30,31,32]. The ability to hold and follow the teacher’s instructions whilst executing movement skills is possible due to a child’s working memory, which is also known as the ‘mental workspace’. Working memory does this, not by acting as a single store of information, but rather by acting with interacting components to manipulate information and enable cognitive flexibility to refocus and shift attention in dynamic environments [30,33].

Physical activity has been explored as a method of enrichment to improve EF skills. All of the EFs work collaboratively and independently during the acquisition of movement skills through the processes of decision-making, planning, problem solving, attending, perceiving, acting and coordinating actions. These will, however, be mediated by the instructional approach of the teacher [34]. A recent review has shown that while overall physical activity levels appear to be linked to higher EF performance, short-term PA interventions only improve EF if the PA has a cognitive component to it [30]. For example, asking children to run 10 laps of the school oval will have less impact (if any) on EF skills compared with a PE class set-up to encourage planning, concentration and problem-solving. In line with this, Tomporowski and Pesce [33] recently suggested that all PA environments need to be mentally challenging and have a focus on skill learning methods in order to lead to sustained enhancement of cognitive functions. They also state that gains from this type of PA will be beyond the gains achieved from exercise programmes that focus on frequency, intensity and dose alone. In the coming section, we will discuss how teachers can manipulate the physical environment within the education setting, according to two prominent theories of motor skill learning, to ensure that the skill learning environment challenges the EF skills of their students.

## 2. Linear Theories of Motor Skill Learning

For much of the 20th century the dominant theories of skill learning were stage theories of learning and information processing theory. Information processing theory [34] suggests that information enters through the sensory system (e.g., visual, auditory, proprioceptive) and, similar to a computer, is encoded and stored in either short- or long-term memory, depending upon the importance of the information. The central nervous system acts as the ‘hardware’ whose function is to order, monitor, select and organise the information, which dictates an internalised prescription of movement, coded as symbolic knowledge structures. Information processing theory postulates a top-down approach to movement with a construct located inside the brain, such as a schema or a mental representation, which is built up or strengthened as a result of the learning process so that a plan of action can occur before a movement emerges [34]. This approach holds with the premise that learning is reflective of the maturation of a mental model and is a gradual linear process. Children become skilled movers through repetition of a skill, while development of a skill progresses through three observable stages of learning: cognitive, associative and autonomous [35]. In the cognitive stage, the learner (child) is overwhelmed by a wealth of information. The child’s full attention is focused on trying to understand the demands of the goal-directed movement and the elaboration of a plan of action (i.e., the movement response). It is characterised by high attentional/cognitive load, whilst execution is effortful, erratic and full of errors. In the associative stage, the child understands the goal-directed movement and tries to gradually reduce the discrepancy between the intended and the actual performance (i.e., reduce variability in the movement) by means of repeated practice, leading to a reduction in attentional demands. In the autonomous stage, the execution of the goal-directed movement or skill typically involves a minimal number of conscious thought processes, whereby accurate and coordinated movements are performed autonomously.

Tomporowski and Pesce [33] propose that there is differential requirement for cognitive skills dependent upon implementation factors, such as the effectiveness of the teacher within the PE environment, related to their ability to identify a child’s stage of learning. To support this, teachers could be trained to identify children in each of the three stages of learning (cognitive, associative or autonomous) and then, prior to the start of the PE lesson, use this knowledge to modify lesson activities using Gentile’s taxonomy (see Table 1) [36]. The 16 categories of the taxonomy would lead the teacher through a logical sequence of potential progressions and require the teacher to be mindful of two main perspectives—the environmental context in which the skill takes place and the function that the movement skill must fulfil. Using Gentile’s taxonomy, a teacher can manipulate the skill into its simplest form, in which the child has a stable base without any object manipulation, in an environment free from distraction. If the teacher believes that a child or class of children have higher competence, they can use Gentile’s taxonomy to create a skill context that is far more challenging, e.g., body in motion, manipulation of an object and environmental factors dictating movement skill responses. Manipulating the environment may include physical changes to the surface used, a change of location for the performance, altering the size of the practice field or the addition of opponents or distractions. This would support the teacher–pupil interface by providing tasks and activities that continuously challenge a child’s EF and, in theory, should see improvements to cognitive function and self-regulation. It may also lead to a long-term change in the way a child processes information, as learning experiences evoke commitment and considerable effort through an integration of the physical, cognitive and affective domains during physical education.

According to Chow et al. [37], linear pedagogy has four main teaching principles. The first underpinning principle is that there is a correct optimal movement pattern for each movement skill. This is based on the idea that there is a movement trace that acts as a reference of correctness to guide a child’s movement. The second key principle is that movement skills are broken down or simplified into key components of a skill for learning, as performing an optimal movement pattern is often beyond the reach of children who are in the cognitive phase of learning. The third key principle is that movement variability is viewed as noise in the system, which the child must reduce in their quest towards mastery of a skill. The fourth, and final, key principle is the focus of attention when learning a movement skill. The majority of research in this area highlights that promotion of an ‘external focus’ generally results in more effective learning and performance of a movement skill [38]. 

## 3. Non-Linear Theories of Motor Skill Learning

The turn of the 21st century has seen the introduction of nonlinear pedagogy (NP), which is drawn from the theory of Ecological Dynamics [39]. This offers an alternative perspective on the learning and development of movement competence and emphasises exploration and discovery [40]. Learning is not linear, nor is it driven by a peripheral agent (top-down approach), but instead, learning occurs through the sudden and abrupt transitions in system organization and is due to self-organising behaviours that evolve through dynamical interactions between system elements. More specifically, this approach suggests that goal-directed movements are the product of the interaction between personal, environmental and task constraints and that movement behaviour is largely self-organised, based on a coupling between perception and action [41]. The first important thing to grasp is that all humans and mammals are viewed as open and complex systems that are always in a process of change, driven by a constant reciprocal interaction between the environment and the individual. The second important thing to grasp is that learning is itself a process of self-organization as a result of intrinsic dynamics transitioning between instability and stability. A key challenge for a teacher is to first appreciate and recognize the observable characteristics of this self-organization of movement behaviour and then to strategically design environments and initiate teaching moments that introduce noise into the learning task, thereby creating an instability that challenges the learner to problem solve functional and adaptive movement solutions [42,43]. In this approach, the perception is direct (as perception action is coupled), and the learners are afforded invitations for action from their environment as they move through it on a moment by moment basis. The concept of affordances therefore highlights the interaction between the environmental features and the functional capabilities of the individual child. A skilled teacher will be able to create accurate scaling of affordances and functional capabilities, allowing distinction between what is possible and what is impossible for movement responses within a specified environment, thus enabling the learner to self-discover and problem solve with a high degree of autonomy and, as such, fully engage all of their EFs.

Two models are proposed to support the teacher in implementing non-linear pedagogy. The first is aimed to help teachers recognise where the learner is currently at on their movement skill journey with respect to a particular skill. This will give the teacher the knowledge and understanding to appreciate the importance of scaling the affordances available in the environment to the child’s functional capabilities or perhaps even slightly beyond these capabilities, inherently creating instability in their movement behaviour. The second is a framework to support teachers during the lesson to indirectly introduce, or reduce, noise (instability/stability) in the system, thus enabling an affordance (goodness of fit) between a child’s functional capacities and environmental features. The first is Newell’s [44] model of movement learning, where the individual is faced with solving the degrees of freedom problem. Newell proposed three observable levels of skill differentiation: coordination, control and skill. Children who are within the coordination stage exhibit movements that tend to be inflexible, rigid and awkward. This is due to the child, at an unconscious level, trying to solve the coordination problem of an unfamiliar goal-directed movement. This coordination problem is solved through freezing out or locking joints and body segments, allowing the child to achieve the movement goal, albeit in a rudimentary form. Through learning, practice and experience, children move to the control stage, which is characterised by movements that are smoother and less rigid, as children seek to discover and explore different movement solutions. Children in the control stage are able to exploit environmental factors to enhance and execute goal-directed movements in an energy-efficient manner that appears almost effortless. Children with low movement competence (i.e., in the coordination stage) tend to adopt the same movement pattern and are less able to identify key affordances for goal-directed movement in their environment. As children discover and explore different movement solutions, their movement competence increases, enhancing their ability to exploit affordances within their environmental context and adapt their movement patterns accordingly in order to achieve the goal. In essence, as children progress through these levels of learning, they become more accomplished problem solvers and more versatile at adapting to changes in environmental demands of the PE lesson. 

During the lesson, the second teaching model, the STEP framework, can also be utilised [45]. STEP can be used to support teachers by manipulating task constraints (space, task, equipment or people) to promote stability or instability in the learner, in order to further support them in the development of functional and adaptable movement solutions. This can be achieved through manipulating the size of the play space, altering the rules of the task, changing the equipment and/or changing the number of people participating in the task. The teacher’s choice of constraint will result in an abundance of new affordances becoming available. One example would be where a child tends to repeat the same movement solutions consistently whilst moving around a play space. He/she could be moved out of their comfort zone and challenged to adapt by the teacher making a rule change to introduce a game of tag. The effect of this is to change the landscape of affordances and take a child/children back to a high level of instability, which would then require a need for exploration of movement solutions.

In brief, NP is defined as the “application of the concepts and tools of nonlinear dynamics” [40] and includes a number of principles that open up two-way lines of communication between teacher and pupil, thereby encouraging the child to problem solve and work towards finding a more functional movement pattern. These principles include representative learning design, the manipulation of relevant constraints (i.e., performer, task and environment), emphasis on ‘task simplification’ in practice designs, promotion of external focus of attention and exploiting the functional role of variability. The net effect of these principles provides the child with autonomy and a need to regulate their behaviours to experiment and create solutions that best answer their individual needs within a given context [46]. The process of searching for alternative movement solutions requires inhibition of previously used solutions and the continuous updating of information held in working memory. Children will need to use the same information but come up with different movement solutions, potentially creating unusual and/or novel solutions, thus developing their cognitive flexibility. This individual difference of functional solutions and communication is an experience that needs to begin in childhood in order to be carried forward to adulthood. Tightly coupled to this is competence support, as the child who has successfully solved their own movement pattern feels a sense of accomplishment that comes from within and is not solely reliant on the feedback and/or praise given by the teacher, as occurs in linear teaching. In addition, teachers utilising nonlinear pedagogy are asking children to find multiple solutions to a movement problem, demonstrating not only their competence but also their creativity. This will drive purposeful decision-making and a strong sense of self-regulation.

## 4. Conclusions

In summary, we have presented here two different PE pedagogies that, with appropriate task and environmental manipulation, can ensure that PE classes are not only physically, but also cognitively challenging. In theory, this should result in improved EF skills and thereby improve self-regulatory behaviours. Further research is required to examine the efficacy of linear and nonlinear pedagogies on children’s physical, affective and cognitive development. In particular, direct comparisons of these two approaches are needed—intuitively, the underlying principles of nonlinear pedagogies appear to lend themselves more to improving cognitive skills, as the learner is more involved in decision-making and problem solving, but this has not been tested. Research into movement learning and control has advanced our knowledge of the physical and cognitive processes involved in the acquisition of movement competence and therefore offers an excellent opportunity to develop a strong theoretical underpinning for a ‘well designed’ primary school PE curriculum that will develop children holistically and result in them leading physically active lives. 

## Figures and Tables

**Table 1:** Example of Gentile’s taxonomy for forehand strike.

	Action Function				
		Body Stability		Body Transport	
		*No Manipulation*	*Manipulation*	*No Manipulation*	*Manipulation*
**Environmental Context**	*Stationary, no intertrial variability*	1. Practice body position for the reception of the ball.	2. Same as step 1, except hold a ball above the target hitting point with other hand.	3. Carry out the entire forehand motion without the ball.	4. Same as step 2, except drop the ball into the hitting zone.
	*Stationary, intertrial variability*	5. Practice the reception of the point of contact position at different levels. The ball could bounce (low, medium and high) in different directions across the court.	6. Same as step 5, except have the ball placed on a hitting tee and alter the level of the tee (low, medium and high).	7. Carry out the entire forehand motion at different levels and approaches to achieve different directions. Move position around the court, stop and simulate the entire forehand motion.	8. Move to different locations in the court, stop and drop the ball into the hitting zone and hit a forehand from a static position in a planned direction.
	*Motion, no intertrial variability*	9. Position for forehand in the return serve position. Learn distance from the baseline. Mimic forehand as partner runs through a serve.	10. Ball is thrown into the right service box, and the ball is caught. The ball is dropped into the hitting zone, and the ball is hit consistently to the left and right service box.	11. Ball is thrown short into the right service box. The player moves from a baseline spot into position to take the shot. See if they can get into position before the ball drops.	12. Ball is thrown short into the right service box. The player moves from a baseline spot into position to take the shot. Do without returns and then add return pace.
	*Motion, intertrial variability*	13. Child starts at various positions on court, based on possible inaccurate shots. Partner repositions based on the player’s various positions. Run through the entire forehand motion again.	14. Same as step 13, except player hits balls to three possible zones: left and right service boxes and between service line and baseline. Partner must adjust to each of the player’s shot locations on the court.	15. Ball is hit over the net to the player. The player runs to position, readjusting when necessary for off-target shots. Partner adjusts to player’s new location, but no shot is made.	16. Same as step 15, except the ball is hit to one of the three zones: right and left service boxes and service box to baseline.
